# Organisation in the Medical Profession

**Published:** 1916-06-03

**Authors:** 


### The Hospital

The Workers' Newspaper of Administrative Medicine and Institutional Life, Administration, National Insurance and Health.

No. 1564, Vol. LX. SATURDAY, JUNE 3, 1916. PRICE ONE PENNY.

### ORGANISATION IN THE MEDICAL PROFESSION.

If we may judge from the declarations of members themselves the one supreme and urgent need of the medical profession is some organisation by which the corporate voice and will of the profession may be expressed. A consciousness of failure in the past and a sense of trials to come combine just now to emphasise this demand. The note is not a new one, but recent events have revived it and strengthened it. To a growing extent the medical practitioner is coming into close association with public bodies and Government offices, and his relation to these organisations has an important effect on his income and livelihood. He brings into the market knowledge and capacity and skill which, judged at first sight, would appear to put him in an absolutely secure financial position; for, so at least it would seem, he has only to combine with his fellows to make his demands effective. Neither the individual nor the community can do without doctors, and a "down-tools" policy, were such a thing conceivable on the part of the profession, would soon destroy all opposition. With so strong an opportunity to apply pressure, it might be anticipated that the professional point of view would be made easily to prevail, and that the monopoly which special knowledge and skill give would be the means of ready victory. But if we may accept what the doctors themselves say, this anticipation is contradicted daily, and, whatever may be the theoretical advantages possessed by the diplomacy of the profession, these are utilised in so feeble or so bungling a fashion that nothing comes of them. Hence the profession, so at least the story goes, is exploited on every hand, and, instead of being one of the most compact and resourceful of organisations, is feeble and helpless before unjust and unreasonable demands. It is said to-day, as it has been said any time these fifty years, that only one thing will provide safety —namely, organisation.

Why, then, is organisation not provided? If we may judge by titles and numbers the profession would seem to have abundant provision. There are Royal Colleges and Royal Societies and Associations in numbers. Journals, some of them devoted almost entirely to medico-political matters, abound. Meetings and committees and reports and recommendations and the amateur adviser are on every hand. Yet matters do not move forward. The machinery or apparatus is certainly here, and it is not from defect in this direction that failure arrives. There are those who cry out for new machines, but they fail to explain why the existing ones are not used. To multiply organisations is neither a politic nor an economic plan, and who will guarantee that the new will be worked more effectively than the old? After all, it is men who translate plans into action rather than those who create ideal constitutions on paper that really count in determining the success of the enterprise.

There are several reasons why it is difficult to secure effective organisation in the medical profession. First, it has to be remembered that to a very large extent doctors work as individuals separate and apart from their fellows. Their daily atmosphere is not of co-operation for a common purpose, and hence they are not accustomed to unite with one another in the prompt pursuit of an end which concerns their material interests. There is further a mechanical hindrance in the difficulty created by irregular hours, with a necessary failure to attend meetings punctually and regularly, and consequent risk of inconsistent or conflicting decisions. In a word, the professional atmosphere and associations do not tend to promote the co operation and union which are essential to the clear definition and persistent application of a well-considered policy. Of course, there are many men who have the qualities necessary to secure these ends, but our remarks here apply less to the individual than to the profession generally and to the circumstances in which members live and think and work. Again, it is necessary to recognise frankly that there are practitioners who dislike so much anything that may be called medico-political agitation or collective bargaining that they will not be associated with any professional organisation. They think such courses inconsistent with the dignity of the profession, or, at least, with their own dignity; and they simply stand aside when anything like corporate action or resistance is proposed. Of course, from the point of view of the organiser and the medical politician these men are sources of weakness, even though individually they may be estimable persons with estimable motives. But this spirit of isolation and of detachment from the sordid things of life is not confined to individuals. It exists also in some of the professional organisations. Of this we had but yesterday a striking example. The powers that be proposed to reduce the fee paid for the notification of any case of infectious disease from half a crown to one shilling. Whether this is just or unjust is not here the question, but in any event the proposal affected general practitioners throughout the country, and it was viewed with much bitterness and some scorn. Yet of all the professional corporations and organisations only one managed to arrange some form of corporate protest and to bring this in an orderly fashion before the representatives of the Government. Of course, the question concerned the general practitioner, and not the consulting physicians and surgeons who form the ruling authorities of colleges and societies which profess to represent the highest professional traditions. But the more completely this contrast is true the more obvious is it that the medical profession has not yet acquired the sense of union and oneness which is the very essence of effective corporate action and movement. Until such a sense is created there will be only a limited success whether for old or for new schemes. There is a great outward difference between the president of a Royal College and an obscure practitioner in the midst of mean streets. But only when the two realise that there is some motive which they possess in common will they successfully organise measures for their interest and protection.

